# Nano-Enhanced Drug Delivery and Therapeutic Ultrasound for Cancer Treatment and Beyond

**DOI:** 10.3389/fbioe.2019.00324

**Published:** 2019-11-22

**Authors:** Priyanka Tharkar, Ramya Varanasi, Wu Shun Felix Wong, Craig T. Jin, Wojciech Chrzanowski

**Affiliations:** ^1^Faculty of Medicine and Health, Sydney School of Pharmacy, Sydney Nano Institute, The University of Sydney, Camperdown, NSW, Australia; ^2^School of Women's and Children's Health, University of New South Wales, Sydney, NSW, Australia; ^3^Faculty of Engineering, School of Electrical and Information Engineering, The University of Sydney, Sydney, NSW, Australia

**Keywords:** ultrasound, nanoparticles, cancer, HIFU, targeted drug delivery

## Abstract

While ultrasound is most widely known for its use in diagnostic imaging, the energy carried by ultrasound waves can be utilized to influence cell function and drug delivery. Consequently, our ability to use ultrasound energy at a given intensity unlocks the opportunity to use the ultrasound for therapeutic applications. Indeed, in the last decade ultrasound-based therapies have emerged with promising treatment modalities for several medical conditions. More recently, ultrasound in combination with nanomedicines, i.e., nanoparticles, has been shown to have substantial potential to enhance the efficacy of many treatments including cancer, Alzheimer disease or osteoarthritis. The concept of ultrasound combined with drug delivery is still in its infancy and more research is needed to unfold the mechanisms and interactions of ultrasound with different nanoparticles types and with various cell types. Here we present the state-of-art in ultrasound and ultrasound-assisted drug delivery with a particular focus on cancer treatments. Notably, this review discusses the application of high intensity focus ultrasound for non-invasive tumor ablation and immunomodulatory effects of ultrasound, as well as the efficacy of nanoparticle-enhanced ultrasound therapies for different medical conditions. Furthermore, this review presents safety considerations related to ultrasound technology and gives recommendations in the context of system design and operation.

## Introduction

Conventionally, ultrasound has been used for imaging and diagnostic purposes. Recent technological advances, in particular in nanotechnology, opened new opportunities for ultrasound to be developed into advanced medical treatments, e.g., ultrasound triggered *in situ* drug synthesis and non-invasive surgery (Ferrara, 2008). Therapeutic ultrasound has already been demonstrated to be effective for prostate, breast and liver cancer ablation, cataract removal, uterine fibroid ablation, phacoemulsification, surgical tissue cutting, treatment of bone fractures, and transdermal drug delivery (Miller et al., 2012). Notably, ultrasound-assisted delivery of drugs has gained increasing attention in recent years as it permits spatially confined delivery of therapeutic compound into target areas, such as tumors (Zhu and Torchilin, 2013). The combination of ultrasound and nano-drug delivery systems removes major limitations of conventional drug delivery systems, including:

– Insufficient uptake and accumulation of nanoparticles by cells (Blanco et al., 2015),
– Limited amount of drug delivered or released from nanoparticles (Du et al., 2011), and
– Targeted specific delivery of drug carrying nanoparticles.

Furthermore, the combination of ultrasound with nanoparticles has a substantial potential to enhance the efficacy of drug delivery and reduce side effects of drugs, through improved transcending of drug carrying particles through physiological barriers—a major goal for advanced drug delivery systems. These physiological barriers include endothelial lining of blood vessels (Thakkar et al., 2012), endothelium of target tissues, tight epithelial cell layers, tissue interstitium, plasma membrane of cells, diffusion through cytoplasm, and ultimately entry into the nucleus via nuclear membrane (if applicable) (Barua and Mitragotri, 2014). In addition to these, the blood–brain barrier (BBB) is a major obstacle for nanoparticle/drug penetration to the brain, which could be overcome by the use of ultrasound (Zhou et al., 2018).

## Barriers for Nanoparticle Drug Delivery to Tumor

Approaches for targeted delivery in cancer may involve systemic administration of chemotherapeutic agents encased in nanoparticles. They may improve the effectiveness of drug delivery and their specificity resulting in targeted drug delivery. To improve targeting, nanoparticles can also be decorated with molecules which specifically recognize and attach to cancer cells. The most commonly utilized tumor specific moieties for targeting are abnormal overexpressed receptors of the tumors. These include endothelial growth factor receptor (VEGFR), epidermal growth factor receptor (EGFR integrin receptor vascular), folate receptor (FR), and human epidermal growth factor receptor 2 (HER2) (Ko et al., 2019). The encapsulation of therapeutic drug molecules in nanoparticles can improve their bioavailability, bio-distribution, and can also improve internalization into the target cell. However, despite recent advancements in the field of nanotechnology, including functionalization with aforementioned targeting molecules, only ~1% of nanoparticles accumulates in tumors (Wilhelm et al., 2016). Therefore, an effective treatment strategy for malignant tumors remains elusive. Very low targeting efficiency could be due to multiple physiological barriers of the tumor architecture (Rosenblum et al., 2018). The first difficulty for nanoparticles just after their intravenous administration, much before they reach the tumor microenvironment is the high chance of getting cleared by blood circulation. It can happen because nanoparticles may be opsonized by blood proteins to be later identified by the cells of the mononuclear phagocyte system (MPS) and finally cleared away from circulation. The nanoparticle populations that avoid clearance by the MPS need to diffuse out of circulation. While nanoparticles are in circulation, they need to effectively accumulate at the endothelial lining toward the tumor micro-environment. Effective extravasation of nanoparticles through the tumor microenvironment represents the second barrier for nanoparticles. The characteristic structure of tumor tissue is distinct compared to normal tissues. The tumor structure usually have abnormal vasculature, show overexpression and presence of high density of extracellular matrix (ECM). The abnormal features of tumor are predominant reasons for inefficient delivery of nanoparticles to tumors. The ECM of tumors consist of a cross-linked network of collagen and elastin fibers, proteoglycans and hyaluronic acid that forms a cross-linked gel-like structure. The highly developed and overexpressed ECM of tumor results in significant resistance to the diffusion of therapeutic nanoparticles through the interstitium. Apart from this, high interstitial fluid pressure (IFP), which is the result of rapid proliferation of cells in a restricted area, the high permeability of tumor vasculature and absence of the lymphatic drainage system declines the force for nanoparticles to penetrate into tumors. These conditions also obstructs the transport and distribution of nanoparticles uniformly into the entire tumor volume. More importantly, the vasculature perfusion within a tumor is quite diverse, leaving several areas with poor vessel perfusion and low blood flow. The situation doubtlessly increases the distance from area over which nanoparticles must travel through to reach target cells, resulting in delivery of nanoparticles to release of therapeutic drug too far from the tumor and its microenvironment. All the distinct pathological features of the tumor profoundly retard nanoparticle delivery, accumulation, and diffusion of nanoparticles uniformly into the tumor leading to inefficient anti-tumor activity of the treatment (Sriraman et al., 2014; Zhang Y. R. et al., 2019).

To overcome the above mentioned barriers there is a need for a technique that can aid in precise delivery of drug at a tumor site and enhance penetration of nanoparticles into tumor volume. Ultrasound-assisted delivery of drug loaded nanoparticles addresses aforementioned limitations by enhancing accumulation and uptake of nanoparticles by cells, as well as by stimulation of drug release only at targeted site. These effects are achieved through various processes like sonoporosis, cavitation, and hyperthermia which occur after the interaction of ultrasound radiation concurrently with cells as well as nanoparticles. Consequently, the use of ultrasound has the potential to improve drug targeting, which in turn may reduce systemic dose of drug required for successful treatment. Therefore, ultrasound-assisted drug delivery may reduce overall treatment side effects associated with drug toxicity and non-targeted delivery (Mullick Chowdhury et al., 2017). Taken together, ultrasound-based drug delivery opens new opportunities for more effective treatments for cancer and other medical conditions.

## Mucosal Barrier

Mucus is a major obstacle for drug/nanoparticle delivery. Presence of mucus in lung, vagina, and bladder pose a challenge for nanoparticle delivery to the diseased area through dense mucosal structure. For example, nanoparticle drug delivery to lung epithelium is challenging due to array of extracellular barriers such as mucociliary clearance and presence of thick mucosal coating around the tissues. In the case of cystic fibrosis a coating of infected sputum is major obstacle for delivery of the nanoparticles to the diseased area. There are a number of ways to overcome these hurdles such as the use of agents which dissolves thick mucus, viscoelastic gels, agents that break tight junctions of mucus for permission of nanoparticles efficiently to the airway epithelium. However, the above mentioned strategies to improve drug delivery through mucous are not effective (Xenariou et al., 2007). In contrast, ultrasound provides a simple and robust solution to overcome the barrier limiting extravasation of therapeutic agent/nanoparticles into mucosal tissues (Chen et al., 2017). The effect can be achieved because ultrasound is associated with energy that can disrupt the mucosal structure allowing nanoparticles to enter the tissues for local drug delivery.

Schoellhammer et al. suggested that low-frequency ultrasound could provide fast delivery of therapeutic agent to the colonic mucosa. It was first explored whether low-frequency ultrasound could improve the delivery of the drugs to the colon tissues in Franz diffusion cells. The findings of the *ex vivo* experiment revealed that treatment with low-frequency ultrasound 20 kHz was able to increase the delivery of dextran (3 kDa) labeled with texas red up to 7-times compared to control. Confocal microscopy images showed that the fluorescent dextran was uniformly diffused throughout the tissues (Schoellhammer et al., 2017). However, until now, the use of ultrasound to improve nanoparticle drug delivery thorough lung mucosal membrane has not been demonstrated. The potential use of ultrasound in transmucosal drug delivery could address the current limitation of low transmucosal nanoparticle/drug delivery for several lung, vaginal and bladder diseases. The presence of the mucus in vaginal epithelium pose an obstacle for obtaining prolonged retention, homogeneous distribution, and successful delivery of drug molecules/nanoparticles in the vaginal tract. Most particulate matter, including nanoparticles, get trapped by mucous through both adhesive and steric interactions due to mucus (Ensign et al., 2012). Mucous barriers also limits the effectiveness of conventional drug delivery systems in treating some bladder conditions including overactive bladder, interstitial cystitis, bladder cancer, and urinary tract infections (Zacchè et al., 2015). In all these situations where mucous forms a physicochemical barrier for drug or drug carrier to reach the target, ultrasound energy could be employed to improve trans-mucosal drug delivery.

## Tumors With Low Enhanced Permeability and Retention (EPR) Effect

Ultrasound could be employed for improving drug delivery to the tumors with low “enhanced permeability and retention effect” (EPR). Some tumors, and typically tumors presenting with an extensive stromal compartment show suboptimal distribution of nanoparticles within the tumor. These tumors are characterized by a dense collagen network and tight perivascular cell coverage. Therefore, the penetration of drugs and drug delivery systems (nanoparticles) into the tumor interstitium is limited. There has not been a huge success so far in developing strategies to improve low EPR of such tumors. Ultrasound could be a useful strategy for improving drug targeting to tumors with low EPR (Theek et al., 2016). Theek, Baues et al. in their study use tumor models (i.e., highly cellular A431 epidermoid xenografts and highly stromal BxPC-3 pancreatic carcinoma xenografts), which both represents relatively low levels of EPR. It was found that the liposome concentrations were increased twice in ultrasound treated tumors as compared to untreated tumors. The effect was observed because of the ability of ultrasound to induce the sonoporation effect. It enhanced the ability of liposomes to penetrate out of the blood vessels into the tumor interstitium. These findings show that ultrasound can improve the efficacy of nanomedicine in tumors treatments which has low levels of EPR.

Taken together, physiological barriers restrain the effective delivery of drugs to targeted tissues. Notably, ultrasound can be used to improve both targeting and effectiveness of drug delivery. Indeed, the ultrasound-mediated drug delivery has substantial potential to improve outcomes of many therapies. However, to move this therapeutic modality to mainstream medicine there is a need to understand effects of ultrasound treatment on cells, tissues, and extracellular matrixes. It is also necessary to understand the ultrasound beam behaviors in the context of complex hierarchical assembly of biological structures; e.g., how the ultrasound beam passes and reflects within tissues and how it interacts with different subtypes of tissues.

## Biophysical Effects of Ultrasounds on Cells

Ultrasound beams of given frequency and intensity can induce various biophysical effects in cells or tissues that are exposed to the energy carried by the ultrasound wave. Well-known biophysical effects of ultrasound on cells are:

- Sonoporation,
- Cavitation, and
- Hyperthermia (Qin et al., 2018).

Each of the effects have different impacts on cell function and can be exploited for different therapeutic applications detailed below.

### Sonoporation

**Figure 1:**
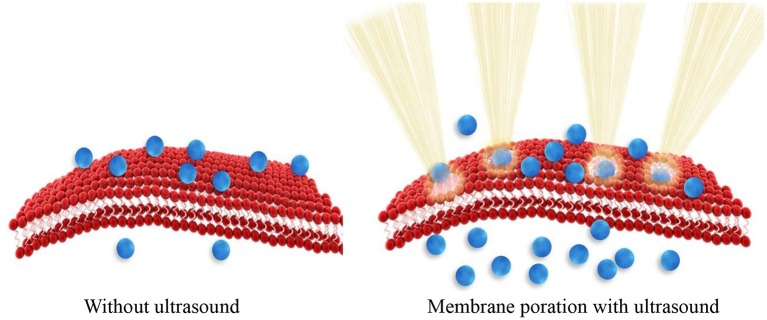
Effect of sonoporosis on cell membrane to enhance nanoparticle drug delivery to cells.

Sonoporation is the process where the size of pores in the cell membrane increases as a result of mechanical impact of ultrasound radiation/energy on cell membrane molecules. Ultrasound physically disrupts the integrity of the membrane assembly causing membrane poration. Tiny pores formed in the membrane enable passive entry of drug molecules or nanoparticles into cells (Figure 1). Consequently, ultrasound can be used to enhance cellular internalization and accumulation of small molecules, genes, and nanoparticles.

**Figure 2:**
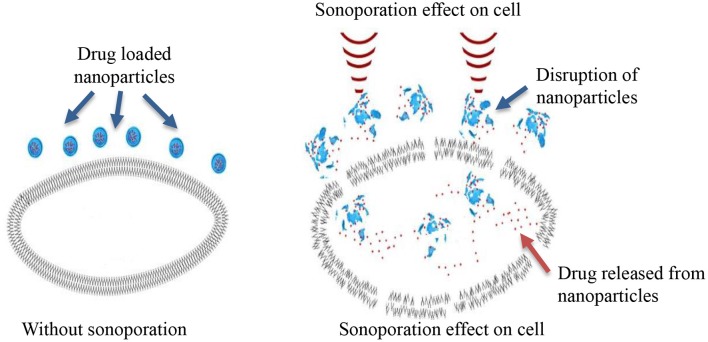
Sonoporesis mediated localized drug delivery to targeted cell.

Sonoporation can be induced in cells through the cavitation process. Cavitation is defined as tiny gas microbubbles being created, developed, vibrated (oscillated), and disintegrated (collapsed) in fluid under the influence of ultrasound radiations. Cavitation occurs for both endogenous and exogenous gas microbubbles. Endogenous gas microbubbles are naturally occurring voids in cell cytoplasm while, exogenous gas bubbles include synthetic gas bubbles, or microbubbles introduced externally in the cellular microenvironment. These comprise of spherical gas- and/or perfluorocarbon-filled cavities and are typically stabilized by a coated surfactant, phospholipid, and synthetic polymer or denatured human serum albumin. Both endogenous and exogenous microbubbles can increase the permeability of cell membranes through the formation of pores on the membrane, which leads to sonophoresis, and regarded as “cavitation induced sonoporation.” Microbubbles can lead to sonoporation once certain cavitation thresholds are achieved. When ultrasound “hits” the microbubbles it induces high-frequency oscillation by absorbing ultrasonic energy. As a result, a fluid jet/shock wave is formed, leading to the perturbation of cell membrane structures. The oscillation and expansion of microbubbles exerts shear pressure on the cell membrane which also enhances permeability of the cell membrane, therefore this process makes cells more accessible to nanoparticles (Figure 2) (Hu et al., 2010). The drug delivery enhanced by the use of microbubbles and ultrasound is regarded as “ultrasound and microbubble (USMB) mediated drug delivery.”

Fechheimer et al. (1986) demonstrated for the first time the ability of ultrasound to induce sonoporation for improved delivery of DNA to mammalian cell. In this study, the suspensions of live slime mold amoebae were exposed to ultrasound and treated with fluorescein-labeled dextran, normally impermeable to cells of its size. There was 40% increase in fluorophore uptake by the ultrasound treated cells and the concept subsequently was translated in mammalian cells for delivering DNA (Fechheimer et al., 1987; Izadifar et al., 2019).

When cells are expose to ultrasound, complex, and often synergistic physical and biochemical processes can take place which include:

1. Increase in intracellular calcium transients
2. Change in plasma membrane potential
3. Production of free radicals
4. Alteration in cell membrane fluidity.

#### Increase in Intracellular Calcium Transients

One of the effects of ultrasound on cell membranes is spontaneous increase in intracellular calcium transient cells (Juffermans et al., 2006; Kumon et al., 2007; Fan et al., 2010, 2012). Increased Ca^2+^ plays a vital part in cell restoration after sonoporation (Hassan et al., 2010). Moreover, calcium influx is shown to stimulate endocytosis in cells (Yang et al., 2019). The process is reversible and a number of studies have demonstrated that intracellular calcium transients have been noticed to enhance immediately and then restoring to equilibrium (Juffermans et al., 2006; Kumon et al., 2007). Jufferman's et al. showed that the increased in Ca^2+^ influx after low intensity ultrasound-exposed microbubbles cause the stimulation of BKCa channels followed by local hyperpolarization of the plasma membrane.

#### Change in of Plasma Membrane Potential

It is well-known that there is a direct relationship between an increase in intracellular Ca^2+^ levels, hyperpolarization and an enhanced uptake of macromolecules through the process of micropinocytosis/endocytosis (Schweizer and Ryan, 2006). These subtle cellular alterations following ultrasound exposure including hyperpolarization of cell membrane were observed when cells were treated with microbubbles. It resulted in the modulation of uptake of large therapeutic agents like plasmid DNA (Juffermans et al., 2008).

Changes in membrane potential can be observed as either hyperpolarization or depolarization of plasma membrane which are associated with sonoporation. Interestingly, it was found that the extent of sonoporation and different intensities of ultrasound can induce diverse effects on the plasma membrane potential. For instance, Wang et al. study found that when cells were treated with low intensity of ultrasound (0.64 W/cm^2^), depolarization in the cell membrane potential was observed. However, when cells were treated with ultrasound of higher intensity (2.1 W/cm^2^), hyperpolarization of the membrane was noticed instantly after irradiation. Their study also detected that the hyperpolarization effect was reversible and returned back to the control level after 180 min (Wang S. et al., 2012).

#### Production of Free Radicals

It is well-established that intracellular reactive free radicals/oxygen species (ROS) are generated during ultrasound cell interaction. The production of ROS is a chemical effect of ultrasound, specifically induced by acoustic cavitation and also known as the sonochemical effect of ultrasound that results in production of ROS (Feril et al., 2008). The production of ROS during the ultrasound interaction with cells and microbubbles is a result of localized increase in temperatures and pressure of several thousand K, and several hundred atmospheres, respectively, of collapsing microbubbles. Local increase in extreme temperature and pressure leads to the decomposition/break down of water vapor into hydroxyl radicals and hydrogen atoms (Riesz and Kondo, 1992). Hydroxyl radicals can form hydrogen peroxide (H_2_O_2_). H_2_O_2_ can initiate different biochemical reactions, i.e., the cascade of production of free radicals. As a result, there is further more formation of free radicals like hydrogen peroxide, singlet oxygen, and superoxide ions. Therefore, measurement of hydroxyl radicals can be used to determine acoustic cavitation quantitatively (Riesz et al., 1990; Wang P. et al., 2012).

Intracellular production of ROS in response to ultrasound play a crucial part in the therapeutic application of ultrasound. For example, low intensity ultrasound produces free radicals that are involved in the membrane permeabilization, which can be employed for molecular delivery of drug, genes and nanoparticles. Thus, ultrasound interaction with tissues produces free radicals that contribute to the production of heat that ablates tissues, and leads to necrosis of cells. ROS can induce cell death in tumors because of their high toxicity and more importantly they also function as signaling molecules for apoptosis in cancer (Hervouet et al., 2007).

A disadvantage of producing high levels of ROS is that can cause some adverse effects like oxidative damage to healthy cells. Other side effects includes denaturation of proteins and damage to tissues. ROS can break DNA (double- or single-strand DNA). The breakage in the DNA backbone essentially occurs between oxygen and carbon atoms, causing DNA fragmentation (Wasan et al., 1996). On one hand, ROS are essential for both cancer and normal cells for their normal function in many processes such as signal transduction (Manthe et al., 2010). Whereas, on the other hand, excessive ROS can lead to carcinogenesis in healthy cells (Ozben, 2007). Unfortunately, the exact quantity of ROS needed to induce tumor cell death is unknown (Ozben, 2007). Excessive apoptosis as a result of ROS production in normal cells can also induce autoimmune disorders, cardiovascular and neurodegenerative diseases, ischemia-reperfusion injury in healthy cells. Therefore, ROS based therapies (including ultrasound) ideally should be specifically destroying cancer cells without causing toxic/harmful effects in normal cells (Wang and Yi, 2008).

Moreover, when ultrasound is used to aid drug delivery, ROS production may decrease the potency of a drug. This is because, free radicals can alter the molecular structure/conformation of the drug, thus reduce the therapeutic effectiveness of the treatment (Zhang et al., 2010).

#### Alteration in Cell Membrane Fluidity

Ultrasound waves have the ability to interact with cell membranes directly or indirectly which results in changes to membrane fluidity that consequently leads to changes in cell function. Giacinto et al. demonstrated that cell membrane fluidity changes after exposure to ultrasound 1 (MHz) using conventional therapeutic ultrasound devices. In their study it was found that the vibration modes of ultrasound can cause reversible change in cell morphology by either compression or stretching of the cell cytoskeleton. The effects were observed because the cellular membrane has a natural tendency to absorb mechanical energy from the ultrasound radiations. It causes expansion and contraction of the intramembrane space upon exposure to ultrasound (Di Giacinto et al., 2019). The transient modifications of cell morphology induced by ultrasound can also result in deformation of the plane of the lipid bilayer of the cell membrane, and the changes in the thickness of the lipid bilayer. The effect consequently can stimulate gated ion channels of the cell membrane and change the electrolyte intracellular distribution of the cells (Wiggins and Phillips, 2004).

Additionally, the membrane fluidity can reduce in reaction to lipid peroxidation. A number of reports have confirmed the reduction of membrane fluidity in different types of cell membranes after of lipid peroxidation induced by ultrasound (Kaplán et al., 2000; Solans et al., 2000; Benderitter et al., 2003). Lipid moieties inside the plasma membrane are one of the targets of ROS. Released ROS after ultrasound interaction with cells often cause lipid peroxidation of plasma membrane. Specially, polyunsaturated phospholipids present in plasma membrane are more susceptible to lipid peroxidation, because of presence of chains in their chemical structure (de la Haba et al., 2013). Lipid peroxidation of the lipids disturbs the cell membrane bilayer structure, changes membrane properties such as membrane fluidity, and also changes the physiological functions of cell membranes which ultimately contributes to cell membrane damage (Catalá, 2009, 2012).

Other effects of ultrasound includes cell body shrinkage, disruption of actin cytoskeleton organization and also cell nucleus contraction (Wang M. et al., 2018).

### Cavitation

Cavitation can be utilized to further enhance the sonoporation effect, and for this purpose microbubbles (exogenous), can be introduced to the cellular microenvironment. Microbubbles are extensively used for diagnostic imaging to enhance the contrast of ultrasound images. However, during imaging ultrasound frequency and intensity is adjusted not to disrupt microbubbles, which otherwise could induce undesired side effects. Microbubbles have also been applied in drug delivery, which take benefit of the capability of ultrasound to disrupt microbubble in selected locations, thus triggering drug release on demand (Unger et al., 2004). Cavitation can be classified into two types depending on how microbubbles collapse in response to ultrasound energy, as stable, and inertial cavitation (Greillier et al., 2018).

#### Stable Cavitation

It is described as a non-linear, sustainable, and periodic expansion and contraction of a gas bubble. During stable cavitation, also called as non-inertial cavitation, the gas pockets present in the liquid oscillate around an equilibrium radius and can persist for long time. This means gas microbubbles can shrink and expand under the influence of ultrasound for long periods of time. The time period of microbubble oscillation lasts until the gas content of the microbubble dissolves into the blood and it is then rapidly cleared through exhalation from the lungs (Khokhlova et al., 2015). The main application of stable cavitation is to alter vascular permeability for increased extravasation (penetration) of nanoparticles and hence improve delivery and deposition of drug/gene/nanoparticle to whole tissues (Kang and Yeh, 2012). It also results in ion channel and receptor stimulation, simultaneously, it can alter cell permeability and action potential of the cell which facilitates drug delivery to cells (Yang et al., 2019).

#### Inertial Cavitation

It can be defined as the violent collapse of bubbles, where microbubbles collapse instantly upon application of ultrasound (Liu D. et al., 2016). The main application of inertial cavitation is to modulate the permeability of individual cells for enhanced delivery of therapeutic agents (genes or drug) at the individual cellular platform (Liu W. W. et al., 2016). Inertial cavitation induces membrane pores of larger sizes in comparison to stable cavitation. The pore sizes can vary from hundreds of nanometers to a few micrometers (Wischhusen and Padilla, 2019). There are three possible mechanisms through which inertial cavitation can alter the permeability of subcutaneous (SC) membrane.

**Modes of inertial cavitation:**

**Figure 3:**
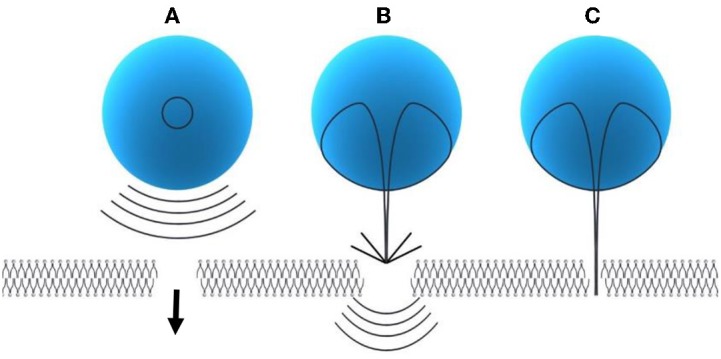
Modes of inertial cavitation [modified from Tezel and Mitragotri (2003), reference number 59 with permission]. **(A)** Effect of spherical collapse of microbubble. **(B)** Effect of an acoustic microjet on SC membrane. **(C)** Effect of microjet entering into the SC membrane.

- Spherical collapse of microbubbles close to the SC membrane radiates shock waves, which has potential to disturb the SC lipid bilayers (Figure 3A)
- Effect of an acoustic microjet on the SC surface. The microjet producing a region about one-tenth of the microbubble diameter influence the SC membrane without entering into the surface of the membrane. The force of the microjet may improve SC permeability by damaging SC lipid bilayers (Figure 3B)
- Microjets may substantially enter into the SC and improve the SC permeability (Figure 3C) (Tezel and Mitragotri, 2003).

**Figure 4:**
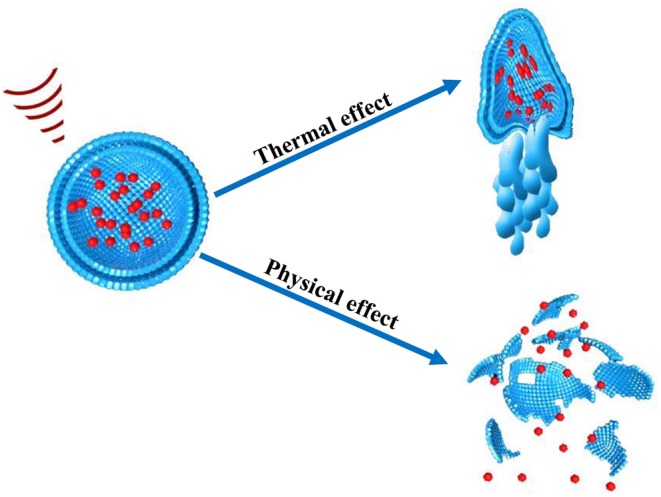
Thermal and non-thermal (physical) effect of ultrasound on nanoparticle.

These effects are also referred as the non-thermal effect of ultrasound. Notably, in non-thermal effects of ultrasound, acoustic pressure, and velocity gradient are generated as a result of shear stress which is produced after the application of ultrasound. The shear stress suppresses the cohesive strengths within the nanoparticles and ultimately ruptures nanoparticles thus releasing their cargo at the site of action. The microjet and microstreaming events has capability to generate temperature increase as well as mechanical stresses within nanoparticle structure (Figure 4). In cavitation extreme stresses such as shockwaves and damaging free radicles are produced which are also responsible disruption of nanoparticles (Husseini et al., 2014).

### Ultrasound-Induced Hyperthermia

Ultrasound induced hyperthermia is the rise in temperature due to the absorption of ultrasound energy by tissue as a result of mechanical compression and decompression. Some portion of the mechanical energy is utilized during friction effects and it ultimately gets transformed into heat. Ultrasound waves cause rotation or vibration molecules in the tissue and these movements results in frictional heat and local hyperthermia in targeted tissues (Kim et al., 2008). With hyperthermia, tissue temperature rises to 40°–45°C which can be up to 60 min, the time period depends on the duration of the ultrasound treatment (Zhu et al., 2019). In general, hyperthermia is utilized in combination with other therapies such as chemotherapy and radiation therapy. Hyperthermia has been successfully used for sensitization of malignant tumors before chemotherapy to treat several types of solid tumors (Hurwitz and Stauffer, 2014).

During hyperthermia, the fluidity of the phospholipid bilayer of the cell membrane changes and results in altered, often increased, permeability of cell membrane for drugs or nanoparticles. The mild increase in temperature can also result in deformation or complete disruption by swelling of nanoparticles structure because of absorption of ultrasound energy. Nanoparticles may eventually burst which helps in enhancing release of therapeutic drug delivery at the desired sight of action. Therefore, increased temperature can act as a “trigger” at the site of action which could be exploited with nanoparticles. Nanoparticles used for such purpose are composed of materials (typically polymers or lipids) which respond to heat, hence called as thermosensitive nanoparticles. Hence, thermosensitive nano/micro particles can be stimulated using ultrasound to enhance the targeted drug release.

For the fabrication of an ideal thermosensitive nanoparticle fundamental characteristics need to be fulfilled, they are as follows:

- Should comprise of phospholipids or polymers in outer structure which are sensitive to heat
- Stable entrapment of therapeutic agent at body temperature
- Instant and enhanced drug release after exposure to heat stimuli and
- Supply of high concentration of drug to plasma while being hyperthermia treatment is applied (Liu D. et al., 2016; Garello and Terreno, 2018).

Another thermal effect of ultrasound is localized thermal ablation, which is achieved with the use of high intensity focused ultrasound (HIFU). Higher intensities of ultrasound (>5 W/cm^2^) are used for local heating of focused tissues for achieving ablation. It is explained in detail as following.

## High Intensity Focused Ultrasound (HIFU)

HIFU is able to induce localized thermal ablation within the body and which is fundamentally different to hyperthermia. Hyperthermia involves heating of tissues up to 40°C, whereas HIFU irradiation induces rapid heating of targeted tissue to temperatures >60°C leading to coagulative necrosis (Chu and Dupuy, 2014). For example, HIFU effectively ablates pancreatic tumors by localized heating of tissues to temperatures as high as 65°C, therefore abolishing the tumor cells completely (Ning et al., 2019). Depending on the intensity, focused ultrasound can be classified into two types: low intensity focused ultrasound (up to 3 W/cm^2^) (LIFU) and high intensity focused ultrasound (HIFU) (over 5 W/cm^2^). Low intensity treatments are typically used for triggering physiological responses to injury to aid healing processes. LIFU is used to stimulate biological reactions that help in healing including: acceleration of soft-tissue regeneration and inhibiting inflammatory responses (Xin et al., 2016). Along with these applications, LIFU is applied for deep-seated tumors (Hayes et al., 2004), as it has been widely reported that low frequency ultrasound beam can propagate into deeper tissues in the body (Hayes et al., 2004).

In contrast, the purpose of HIFU treatment is to selectively destroy tissues through localized heating known as ultrasound induced thermal ablation (Hayes et al., 2004). For HIFU, a broad range of ultrasound frequencies are used from about 20 kHz up to hundreds of MHz. Frequencies less than a few hundred kHz are regarded as low frequency ultrasound, whereas frequencies equal to and higher than 1 MHz and are regarded as high frequency ultrasound (Erriu et al., 2014). In HIFU, hyperthermia results from focusing of high intensity ultrasound beam on selected areas for a certain amount of time in one area (van den Bijgaart et al., 2017). Hyperthermic cell ablation can be very precise since an ultrasound beam can be focused on a small area ~1 mm in diameter and about 10 mm in length (Emfietzoglou et al., 2005).

In China and Korea, HIFU has been extensively utilized for the treatment of cancer and other conditions since the 1990s. More recently, National Institutes of Health (NIH) (2013) recommends HIFU as an auxiliary therapy for unresectable pancreatic ductal adenocarcinoma (PDAC) (Ning et al., 2019). There has been substantial successes with the application of HIFU for non-invasive “surgery” that allows precise ablation of solid tumors, such as breast cancer, prostate cancer, hepatocellular and pancreatic carcinoma, uterine fibroids and bone malignancies (Wu, 2006). The most important benefit of HIFU is that it is less harmful than a surgical procedure. It does not require opening and cutting the body surface to access a tumor site, thus eliminating the need for anesthetics. It also minimizes surgery related biological waste, morbidity, mortality, hospital stay length, and expenses. Overall it can improve quality of life for cancer patients (Cirincione et al., 2017).

### HIFU-Induced Immunomodulation

The healthy immune system has the capability to identify a broad range of pathogens and cancer cells. A compromised immune system in cancer patients is one of the primary issues which is responsible for the advancement and promotion of cancer. Cancer cells develop various “strategies” to fully escape immune surveillance (Costello et al., 1999) by releasing cytokines (with immuno-suppressive capabilities) and by depleting the tumor associated antigens (TAAs) (Lindau et al., 2013). Therefore, in order to hinder recognition by T lymphocytes (immune cells) of neoplastic cells, cancer cells down-regulate the expression of tumor antigens. Moreover, cancer cells can deactivate effector T lymphocytes by releasing immuno-suppressive cytokines (Konjević et al., 2019). The important function of anti-tumor immunity in cancer is the specific identification and destruction of cancer cells by immune system of the patient. For achieving the effect, cancer cells need to indicate tumor-associated antigens (TAAs) and produce a tumor specific immune response. Unfortunately, it doesn't happen in cancer because of a compromised immune system. Therefore, some of the immunotherapies focus on the modulation of the immune system through adoptive T cell transfer, T cell checkpoint blockade, or vaccination. These immunomodulatory strategies are rising as a potential and effective therapeutic approach with clinical benefit for cancer patients (Ayoub et al., 2019).

Interestingly, HIFU, which can induce both mechanical effects and hyperthermia, stimulates physiological responses of the immune system—immunomodulation. The effects of HIFU further extends benefits and applications of HIFU in cancer treatment. Pre-clinical and clinical research have showed that HIFU leads to modulation of long-term systemic host anti-tumor immunity (Hu et al., 2007; Wu et al., 2007; Zhou et al., 2008). HIFU-induced immunomodulation is likely to support the removal of cancer cells left at the local treatment site, thus suppressing local recurrence, and distant metastasis in cancer patients with original dysfunction of anti-tumor immunity (Cirincione et al., 2017).

The immunomodulatory function of HIFU can be explained by the following theories:

1. After HIFU-induced thermal ablation, tumor debris remains, and TAAs are released in response to the ablation. These remaining debris and released TAAs can generate hazard signal over-expression, which in combination with each other function as tumor vaccines and enhance tumor immunogenicity.
2. HIFU stimulates a Th1 type cell responses, which gives rise to substantial modulation in “cell-mediated immunity.”
3. HIFU therapy works on balancing of cancer-induced immuno-suppression in the surrounding tumor environment (den Brok et al., 2004; Toraya-Brown and Fiering, 2014; Cirincione et al., 2017; van den Bijgaart et al., 2017; Yang et al., 2019; Zhu et al., 2019).

## HIFU Use for Different Tumor Types

### Uterine Fibroids

It has been discovered that cancer cells are more sensitive to heat than normal cells (Peek and Wu, 2018). For this reason, the use of ultrasound provides the opportunity for a non-invasive technique to treat various cancers of both primary solid tumors and metastatic disease. The first approved HIFU device by the FDA was in October 2004 for the treatment of uterine fibroids (Jain et al., 2018).

Uterine fibroids are among the most common tumors occurring in the female genital tract, however due to their benign nature they often grow undiagnosed for many years and cause serious health problems such as prolonged menstruation, abdominal pressure leading to pain and other obstetric complications. The standard of treatment for many years has been surgical management which leads to a full hysterectomy thus removing the choice of fertility for these women.

An evaluation of HIFU ablation for uterine fibroids in 2017 established HIFU caused considerably less morbidity than surgery with similar results with quality of life. This clinical study reported 1353 women with uterine fibroids receiving HIFU for 472 hysterectomies and 586 myomectomies (Chen et al., 2018). Another recent study by Łozinski et al. in 2019 attempted to see the effects of HIFU treatment whilst preserving fertility. The results showed that HIFU does not impair ovarian function nor negatively effects the ability to conceive due to its minimally invasive nature. It was recommended that this therapy should only be considered as an alternative treatment until more data is established (Łozinski et al., 2019).

Such clinical studies are displaying the prospective uses of HIFU and specifically in the use of cancer treatments.

### Brain Cancer

HIFU with guided systems are being heralded as a “disruptive technology” due to the possibility of overturning standard technologies used for therapy. This is because providing therapy to discrete brain targets whilst keeping an intact skull has been always been a therapeutic goal (Meng et al., 2017). In the brain, ultrasound has been used for the ablation of glioblastoma and functional neurosurgery (Bradley, 2009). Apart from ablation properties, ultrasound systems are proving advantageous in brain tumor surgery due their real time imaging and 3D-navigation properties in the head region allowing intra-operative neuro-navigation (Wei et al., 2013).

Another application of ultrasound in the treatment of brain cancers is through chemotherapy delivery. Many chemotherapies have low bioavailability in the brain and have even showed neurotoxicity due to non-specific brain uptake. Ultrasound mediated BBB disruption to administer chemotherapy has received multiple preclinical studies and a pilot trial has started to investigate the delivery of chemotherapeutic agent temozolomide (Meng et al., 2017). Further details about drug delivery across BBB below.

### Breast Cancer

A predicament many surgeons currently face when removing breast tumors is their inability to visualize the tumor in determining clear margins between healthy and malignant tissue. This issue leads to higher rates of reoperation to excise the residual tumor allowing for ongoing complications. HIFU ablation with guidance using ultrasound or MRI has the potential to resolve this problem due to its non-invasive nature and ability to plan through visualization and real time monitoring (Peek et al., 2015).

Peek et al. summarizes the various HIFU devices currently in use for application in breast and outlines several clinical trials comparing ultrasound and MRI image guidance with positive results (Peek and Wu, 2018). In the 2015 meta-analysis, 46% of patients resulted in complete ablation and 30% reported near complete ablation with <10% residual tumor. The treatment times ranged between 78 to 171 min and the most common side effect of 40% was pain (Bea et al., 2018).

These results show that HIFU is a viable surgical technique for removal of breast cancer. However, the issue with long treatment times and reduction of pain is one that should be strongly considered. The HIFU technique should look at solutions to reduce adverse effects such as pain and move to larger populations of clinical trials to validate these positive results.

### Pancreatic Cancer

For many pancreatic cancer patients, the time of diagnosis is already too late and inoperable due to locally advanced disease or metastasis. Ablative HIFU therapy has provided an alternate primary and palliative therapy for pancreatic cancer. Endoluminal HIFU transducers are in pre-clinical development and will allow endoscopic placement inside the stomach or duodenum to be precisely adjacent to the pancreas. This will ensure greater lesion targeting and minimizes risk of damage to soft tissue in neighboring areas (Maloney and Hwang, 2015).

Apart from ablative primary therapy HIFU is being applied for greater drug targeting as current traditional chemotherapy drugs are poorly effective in penetrating fibrotic and hypovascular stroma of pancreatic adenocarcinoma (Maloney and Hwang, 2015). Preclinical and clinical studies show the use of HIFU to deliver liposomes into pancreatic tumors applying the process of sonoporation. The study administered pancreatic cancer patients with a combination of gemcitabine and ultrasound with microbubbles causing a survival rate of 17.6 vs. 8.9 months with gemcitabine alone. The sonoporation technique indicated that HIFU can be used to enhance penetration into the pancreatic tumor interstitium (Rix et al., 2018).

### Prostate Cancer

In the case of prostate cancer, HIFU has been used for treatment for a long period of time with both established systems as well as consistent developments. The HIFU transducer is placed within the rectum or urethra and the cancer is ablated via guided-HIFU therapy. Recently, this procedure is also being used for partial prostate gland ablation (Rix et al., 2018). Juho et al. evaluated the therapeutic response and complications of HIFU for patients with localized prostate cancer. The study evaluated clinical outcomes of 29 patients who received HFU as first-line treatment and results showed a 100% survival rate on the 24.6 month follow up with ~20% biochemical recurrence and similar rate for disease progression. Overall, this study confirms HIFU as an alternative therapy in patients with localized prostate cancer with a low complication at follow up in the short term (Juho et al., 2016).

### Liver Cancer

It is estimated more than 80% of patients with hepatocellular carcinoma (HCC) are poor candidates for curative surgery due to advanced underlying cirrhosis. A minimally invasive method such as HIFU is gaining prominence for this reason (Maloney and Hwang, 2015). Studies of HIFU use in hepatocellular carcinoma and secondary liver metastases in human clinical trials have been published with significant promise in HIFU treatment of hepatic malignancies (Dubinsky et al., 2008).

Young et al. describes a case study of a patient with liver metastases where the tumor was not resectable and had received systemic chemotherapy. The patient was treated with a single HIFU session with complete lesion ablation under general anesthesia. The outcome of this session showed a complete remission of the metastatic liver mass (Sung et al., 2008). HIFU therapy for liver lesions remain in at early stages but may offer solutions to current therapeutic barriers (Maloney and Hwang, 2015).

## Ultrasound Induced Mechanism of Drug Delivery in Cancer

Cancer is a leading cause of death worldwide. Systemic chemotherapy is the primary therapy used to treat various types of cancers, but it is associated with undesirable side effects and have shown unsatisfactory tumor responses. Poor tumor responses to chemotherapy arises because of major obstacles for the diffusion of anticancer drug to the tumor site. The main obstacles include the diverse and unorganized tumor vasculature, abnormal and atypical blood flow, and high interstitial pressure within the tumor tissue. It results into low and heterogeneous uptake of nanoparticles in tumor tissue which are one of the main problems for successful cancer therapy using nanoparticles. The insignificant response of tumor to therapeutic agents and nanoparticles have strongly indicated the need for developing a new strategic approach for improving targeted drug delivery to tumors and minimizing systemic side effects of the treatment at the same time.

Ultrasound has been utilized to improve the delivery of therapeutic agents for the past three decades; it includes chemotherapeutic drugs, proteins, and genes (Pitt et al., 2004). There are various ways by which ultrasound improves the efficacy of treatment, specifically by:

- Triggering the release of the drug from nanoparticles,
- Promoting uptake and accumulation of nanoparticle in cells,
- Enhancing the penetration of nanoparticles in tumors.

### Triggering the Release of Drug From Nanoparticles at Target

The exact mechanism of how ultrasound disrupts nanoparticles and how they react to an ultrasound beam to stimulate nanoparticles is still not fully elucidated (Zhou et al., 2014). There are two possible mechanisms of disruption of nanoparticles by ultrasound i.e., thermal effect and non-thermal effects. Thermal and non-thermal mechanisms could also work as an adjunctive stimulus during drug delivery. Therefore, multi-mechanisms such as thermal and mechanical effects of ultrasound combines and act simultaneously for disruption of nanoparticles (Zardad et al., 2016). Drug delivery systems can be fabricated to react either to the increased temperature or to the mechanical effects of an ultrasound beam, or to both (Schroeder et al., 2009b).

#### Localized on Demand Triggered Drug Release

**Figure 5:**
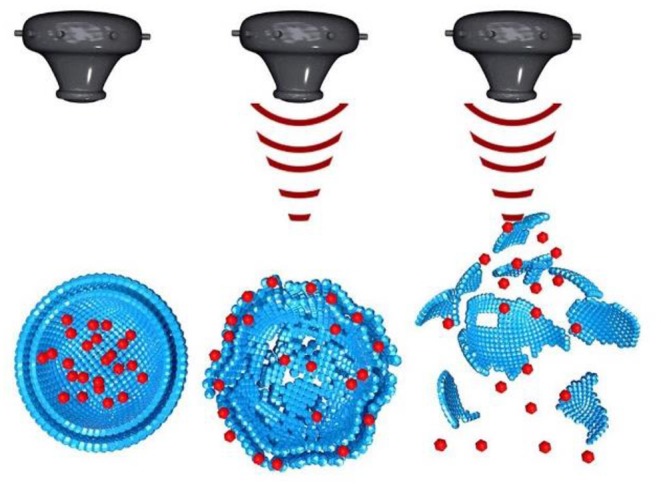
Ultrasound induced disruption of nanoparticles [modified from reference Wang X. et al. (2018), reference number 114 with permission].

The universal drawback of cancer is unspecific drug delivery of chemotherapeutic drug on healthy cells. One of the distinctive characteristics of ultrasound is that it can be focused. The effect is similar to the focusing of light through a lens which can burn paper if focused for long time. In a same manner ultrasound can be focused using acoustic lens to an object in liquid medium. The focus of the ultrasound can be adjusted as small as a few cubic millimeter (Mason, 2011). This characteristic of ultrasound can be utilized for focusing an ultrasound beam electronically and with accuracy on soft tissues (such as tumor) for thermal ablation and on to nanoparticles for their disruption (Figure 5) to achieve localized drug delivery to tumors (Wang X. et al., 2018).

In a summary, ultrasound enables the disruption of drug carrying nanoparticles and release of drug locally at tumor sites. Therefore, the combination of ultrasound and ultrasound-sensitive nanoparticles can enhance delivery of drug from nanoparticles and selectively release drug within the focal point of ultrasound. It can synergistically reduce the amount of dosage required and facilitate selective tumor targeted drug delivery to avoid undesirable side effects on healthy cells.

### Promoting Uptake and Accumulation of Nanoparticle in Cells

A major problem in cancer therapy, where drug is delivered with the help of nanoparticles, is the very low and uneven uptake of nanoparticles in tumor tissue. According to Wilhelum et al. only about 0.7% of the intravenously administered dose of nanomedicine is delivered to the tumor (Wilhelm et al., 2016). Nanoparticle uptake of the tumor can be increased if ultrasound is applied, which is attributed to the enhanced transport of nanoparticles to the tumor. Furthermore, microbubble (which are gas bubbles stabilized by coating of surfactant, polymer, or phospholipids) in combination with ultrasound can be utilized to enhance transport of nanoparticles/drug toward tumor. Upon irradiation with ultrasound, the microbubbles which are administered in the bloodstream will oscillate and induce mechanical forces on the blood vessel wall. The externally received mechanical energy from ultrasound can enhance the transport of nanoparticles and drugs across the capillary wall to enter the extracellular matrix of tumor.

### Enhancing the Penetration of Nanoparticles in Tumors

The efficacy of treatment for any disease is determined by localized concentration of therapeutic agent and duration *in situ*. For instance, in cancer drug delivery into tumor tissue is achieved by penetrating through the capillary wall or through the tumor stroma to tumor cells, resulting in tumor cell death. Unfortunately, in current chemotherapy for cancer, the localized concentration of drug within tumor is low, and the drug concentration in bloodstream is comparatively high. It leads to toxicity to healthy cells, causing unwanted side effects (Wei et al., 2019). Therefore, enhanced drug penetration in solid tumors and reduced possible side effects on healthy cells remain a major goal in cancer chemotherapy. The problem arises because of overexpression of dense extracellular matrix (ECM) by tumor cells. ECM acts as a steric barrier to transport and distribution of nanoparticles throughout the tumor (Chauhan et al., 2011). This issue can be addressed by using ultrasound, as mechanical energy associated with ultrasound physically “pushing” nanoparticles into the tumor matrix, enhancing accumulation and deeper penetration of the particles within diseased tissue. The major limitation of current drug delivery systems is that they can travel only a few μm from main blood capillary inside the tumor.

For example, nanoparticles such as liposomes fail to distribute deeper into dense tumor mass (Emfietzoglou et al., 2005). It was studied in 2006, by Dreher et al. performed expanded study on penetration of dextrans of 3.3–2,000 kDa molecular weights into solid tumors. Their study revealed that diffusion of 40–70 kDa dextrans was mostly confined to 15 μm region from the closest capillary. The permeation of 2,000 kDa dextrans was constricted to only a 5 μm from the nearby vessel wall (Dreher et al., 2006).

Kostarelos et al. (2004) also demonstrated that liposomes mainly concentrate close to the tumor vasculature, and are unable to penetrate deeper into dense tumor mass (Poh et al., 2015). Poor permeation results in inadequate exposure to antitumour therapeutics and contributes to development of chemo-resistance and increased metastasis. Ultrasound enhances the penetration of nanoparticles into tumor tissues to entirely and adequately expose tumor to therapeutics. Taken together, ultrasound-based drug delivery enhances efficacy of cancer treatment by improving the cellular uptake of nanoparticles, enhancing penetration of nanoparticles in tumor, and increasing the therapeutic delivery of the drug (by disruption of the particles on the site of action) (Hare et al., 2017).

## Ultrasound Interaction With Nanoparticles

When ultrasound radiation propagates through the body, it can interact with cells and engineered nanoparticles used to deliver drugs which are circulating in the blood stream or reside in the extracellular matrix. Since the ultrasound radiation carries a substantial energy, it simultaneously interacts with cells and nanoparticles. The effects of interactions of ultrasound with nanoparticles and cells are favorable for drug delivery in cancer and synergistically enhance the effectiveness of the treatment. Moreover, ultrasound drug delivery with nanoparticles has a capability to overcome limitations associated with current cancer therapies.

Since ultrasound has the ability to interact with nanoparticles, it is possible to design drug-carrying nanoparticles that in response to ultrasound break open and release a therapeutic payload. Therefore, ultrasound can be used to trigger drug release on demand from nanoparticles known as ultrasound-mediated drug release. Besides triggering the drug release through the disruption of the nanoparticles, ultrasound can be used to modulate intracellular pressure, as well as to induce acoustic fluid streaming, cavitation, and local hyperthermia. In fact, each of these mechanisms can be used to trigger drug release from a carrier which requires designing nanoparticles that are sensitive to one or more of these triggers (Sirsi and Borden, 2014). For example, co-polymer based nanoparticles such as ultrasound-sensitive block copolymer micelle like poly ethylene glycol (PEG) and poly propylene glycol (PPG) (Li et al., 2016) can breakdown under the influence of ultrasound to release bolus of drug precisely at the targeted tissues (Wang et al., 2009).

Since the outcomes of the interactions between ultrasound and nanoparticles are different, e.g., disruption or melting of nanoparticles (Figure 4), it implies that different cellular pathways will be affected by each of the interactions. It offers substantial benefit because it is possible to design nanoparticles which act on a specific pathway that is complimentary to drug action; e.g., thermosensitive nanoparticles for heat-controlled drug release. When two treatment modalities are combined it is expected that the efficacy of the treatment will be improved. For example, combination of hyperthermia, and chemotherapy leads to increased effectiveness in inducing cell apoptosis (Boissenot et al., 2017).

Furthermore, ultrasound can improve the accumulation of drug- encased nanoparticle in target tissues. The enhancement is attributed to the mechanical energy carried by ultrasound radiation that physically pushes nanoparticles within body. The physical push enhances the transport of nanoparticles toward ultrasound-exposed tissues and results in localized accumulation and increased cellular uptake of nanoparticles (Watson et al., 2012).

### Types of Ultrasound-Sensitive Materials and Nanoparticles

It is well-established that ultrasound can induce the degradation of polymers to lower molecular weight. Earlier research revealed that ultrasound induce depolymerization is a non-random process. Generally, the polymer chain separation occurs mostly at the chain midpoints, and larger molecules degrade the fastest. Another noticeable characteristic of ultrasound induced degradation is that the molecular weight reduction is the result of splitting of the weakest chemical bond in the chain. Reich et al. demonstrated that ultrasound intensity of 40 W and above can substantially reduce molecular weight of poly lactic acid (PLA) and poly lactic-co-glycolic acid (PLGA), even at short treatment duration of 30 or 20 s. EI-sherif et al. also reported that the high frequency ultrasound of 5–10 MHz can induce decomposition of PLGA polymer under a high frequency ultrasound. Therefore, use of ultrasound can be an effective method for designing of required polymers. The exact mechanism of ultrasonic degradation remains poorly understood. However, it is now established that the high shear fields and immediate generation of hot spots after ultrasonic cavitation are basically accountable for polymer degradation. It is also considered that at the level of cavity collapse created by ultrasound beam, friction forces and shock waves form the stresses on the surface of a polymer chain, and also into the polymer coil. It results in chain bond decomposition in large molecules in liquid (Xia et al., 2016). Table 1 summarizes the use of nanoparticles in combination with ultrasound to enhance efficacy of the treatment.

**Table 1:** Use of nanoparticles with ultrasound to enhance efficacy of the treatment.

**Nanoparticle categories**	**Features**	**Reference**
Micelles	•Able to preferentially accumulate in tumor cells due to enhanced permeability effect • Demonstrated use for sonodynamic therapy to generate reactive oxygen species at tumor site	Horise et al., 2019
Liposomes	•Easy preparation • Good biocompatibility • Low toxicity • Can modify surface by PEGylation to increase circulation time	Mangraviti et al., 2016
Solid lipid nanoparticles	•Can hold a high payload • Can hold both soluble and insoluble actives • Greater stability than liposomal and polymeric nanoparticles • Good biocompatibility	Özdemir et al., 2019 Sadegh Malvajerd et al., 2019
Mesoporous silica nanoparticles	•High stability • Biodegradable • Biocompatible • Can also be used as contrast agents for theranostic ultrasound applications • Can be used to sensitize tumors to effects of hyperthermia	You et al., 2016
Perfluorocarbon containing nano-/microparticle	•Nano-sized with liquid-gas phase transition • Can be used to form microbubbles to form cavitation effect with HIFU • Can be encapsulated in lipid or polymer materials	Zhang Y. et al., 2019

#### Micelles

Micelles are self-assembled colloidal structures made up of amphiphilic molecules in an aqueous solution. They are formed by a hydrophilic outer shell and are able to avoid engulfment by the immune system (Hanafy et al., 2018), making them very useful for targeted drug delivery especially for cancer therapy. Polymeric micelles have received considerable interest due to their ability to penetrate the vasculature of tumors and have shown both high drug capacity and good efficiency in patients with malignancies of breast and lung.

Micelles sensitive to biological stimuli such as ultrasound offer a great opportunity for drug delivery as controllable systems (Hanafy et al., 2018). Recently, sonodynamic therapy with anticancer micelles and high intensity focused ultrasound was investigated in canine cancer. This study by Horise et al. was able to confirm the anticancer efficacy of their method and showed its potential to become standard therapy in human cancer (Horise et al., 2019). This study and others indicate the potential of ultrasound waves to disrupt micelle nanocarriers allowing diffusion of drugs in cancer targets.

#### Liposomes, e-Liposomes

Thermosensitive liposomes (TSLs) encapsulate a hydrophilic drug within a core surrounded by a lipid bilayer. TSLs take advantage of the enhanced retention and permeability effect in tumors due to their nanosize and ability to release an encapsulated drug in response to hyperthermia caused by ultrasound (Jain et al., 2018). For example, the study done by Yudina and Moonen (2012) outlines the use of doxorubicin-loaded and paclitaxel-loaded TSLs (Yudina and Moonen, 2012). The drug-loaded TSLs were externally triggered in response to being treated with ultrasound through hyperthermia near their phase transition temperature. This study demonstrated improved effectiveness in preclinical trials for drug bioavailability and anti-tumor effect. The hyperthermia range for TSLs are ~40–45°C and it has been reported that drug delivery using TLS could be enhanced by using prolonged hyperthermia for up to 2 h (Elhelf et al., 2018).

#### Solid Lipid Nanoparticles (SLNs)

SLNs are an attractive means of drug delivery owing to their ability to combine the advantages of both polymeric nanoparticles and lipid structured nanoparticles such as liposomes. This is because they are suitable to hold a high payload of both soluble and insoluble actives; their size range is flexible (50–1,000 nm); greater stability compared to liposomal and polymeric nanoparticles; and are safe within the body even for an extended period due to their low toxicity. The SLN can be made to have a drug enriched core surrounded by a solid lipid shell or the shell could contain the drug and the core be made of solid lipids (Özdemir et al., 2019; Sadegh Malvajerd et al., 2019). For these reasons SLN are very suitable nanocarriers for drug targeting such as for penetrating the BBB (Sadegh Malvajerd et al., 2019).

The use of solid lipid nanoparticles with ultrasound triggered drug delivery is a novel technique. The principles of delivery with ultrasound is applicable to this category of nanoparticles. Timbe et al. describes guided ultrasound delivery of cisplatin-loaded brain penetrating nanoparticles (Timbie et al., 2017). The study hypothesized that ultrasound could enhance the efficacy of drug-loaded brain penetrating nanoparticles for the treatment of glioblastoma. The study was able to show a marked improvement of delivery and distribution when compared to the control. The novel use of ultrasound triggered SLN as nanocarriers are an attractive option and yet to be explored in great detail.

#### Mesoporous Silica Nanoparticles

Biocompatible mesoporous silica nanoparticles (MSNs) are inorganic nanosystems which have the ability to produce high performance molecular imaging, drug delivery and biosensors. In 2018, Wu et al. demonstrated doxorubicin encapsulated MSN could be accurately delivered into brain tumors that were concurrently triggered by focused ultrasound exposure and resulted in significant inhibition of orthotopic brain tumor progression (Wu et al., 2018). Hollow mesoporous silica nanoparticles (HMSNs) have been employed as carriers of thermos-sensitive perfluorohexane (PFH), a hydrophobic chemotherapeutic agent to act synergistically with HIFU cancer surgery (Chen et al., 2014). The mesopore channels in the shell make it possible to encapsulate and continuously release the thermosensitive PFH due to local temperature rise induced by HIFU. The procedure of fabricating PFH loaded HMSNs is to first use etching protocols to produce the HMSNs, then PFH is loaded into the pore network and inner cavities using a mild infusion procedure. After administration and exposure to HIFU, the liquid PFH is converted into small bubbles which swell and merge upon accumulation in the targeted tumor tissues (Wang X. et al., 2012). This process makes use of HIFU's mechanical and acoustic properties to enhance ablation at tumor sites.

#### Perfluorocarbon Containing Nano-/Microparticle

Perfluorochemicals (PFCs) are inert and highly fluorinated organic compounds that can dissolve large volumes of respiratory gases including oxygen, O_2_ (Li et al., 2018). High gas solubilities of PFCs forms the foundation for an abiotic form for intravascular oxygen delivery. PFCs are characterized by a unique feature of chemical and biological inertness, and the gas contents excreted easily as a vapor by exhalation when administered in body. PFCs are intensely hydrophobic and lipophobic in nature (Riess, 2005). Therefore, PFC liquids are not soluble with aqueous phase, including blood, but they can be formulated as emulsions and can be administered into the bloodstream in a safe manner. PFC emulsion formulations are presently being tested in clinical trials as an alternative means for intravascular respiratory gas-carriers and tissue oxygenating fluids, regarded as ‘blood substitutes' (Krafft and Riess, 2007; Chen et al., 2013). PFC compounds are responsive to ultrasound, it was first demonstrated by Apfel, in his pioneering work for more than two decades proving that the specifically fabricated perfluorocarbon droplets can be transformed into microbubbles after ultrasound application. Kripfgans et al. also demonstrated that micrometer-sized PFP droplets can be vaporized into gas bubbles with the application of ultrasound (1.5–8 MHz), the process is regarded as “acoustic droplet vaporization” (ADV) (Rapoport, 2012). It leads to formation of microbubbles that can act as the contrast agents for diagnostic ultrasound imaging. Perfluorocarbon can also be employed for several other biomedical applications such as lung surfactant replacement and ophthalmologic aids. Many other colloidal PFC formulations of are being tested for molecular imaging using ultrasound or magnetic resonance, and for targeted drug delivery.

## Miscellaneous Applications of Therapeutic Ultrasound

### Drug Delivery Through Blood Brain Barrier

**Figure 6:**
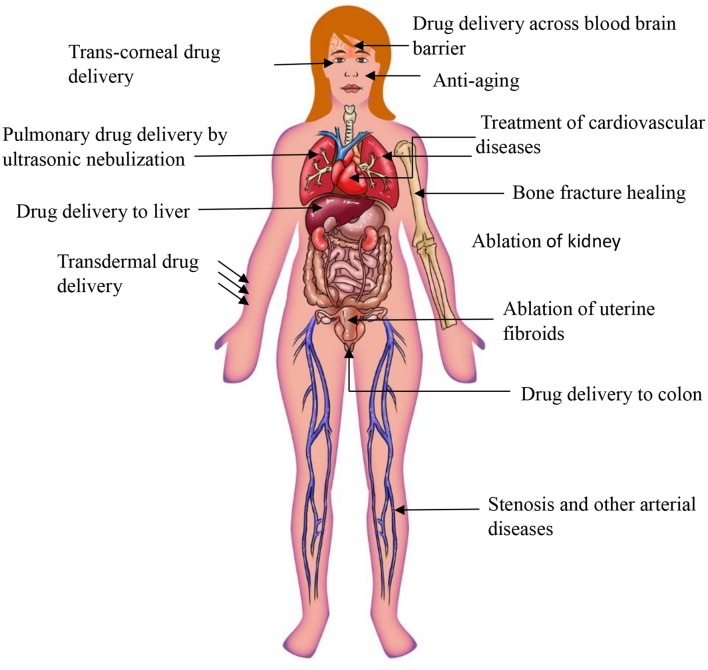
Miscellaneous application of ultrasound.

The HIFU method involves the selective and localized disruption of the BBB to increase permeability (Figure 6). Typically, low frequency ultrasound waves have been employed using perfluorocarbon gas microbubbles which have been intravenously administered. The microbubbles assist in the opening of the BBB by passaging through capillaries and expand and collapse due to mechanical forces of the ultrasound. Results have shown the process is safe and the disruption to the BBB is reversible, lasting up to 4 h with no neuronal damage (Etame et al., 2012). The 2002 Mesiwala et al. study was able to successfully show that HIFU can transiently open the BBB without causing associated parenchymal damage in animal models (Mesiwala et al., 2002).

The most important consideration of HIFU in its application to BBB is that as the frequency increases the degree of tissue attenuation decreases which can lead to skull heating and distortion. The risk of tissue damage is also amplified as the degree of BBB permeability is increased. Therefore, even though an ideal frequency of 200 kHz to 1.5 MHz has been suggested for transcranial use, it is not comprehensively applicable and also depends on factors such as beam pulsing and dosage of microbubbles. Thus, considerable efforts still need to be made in establishing protocols and safety evaluations for use in BBB permeability for neurological applications (McMahon et al., 2019).

### Alzheimer's Disease

As with brain tumors, overcoming the limitation the blood brain barrier could reform many central nervous system therapies including Alzheimer's disease. Alzheimer's disease treatment is currently very limited and reversing the progression of the disease is difficult. One of the principal mechanisms of this disease is the accumulation of amyloid plaques which results in loss of neurons (Chang and Chang, 2017).

A weekly study showed non-invasive opening and HIFU treatment of the BBB at targeted bilateral hippocampal areas in mice models allowed accumulation of endogenous antibodies and reduced amyloid plaques by ~20%. This HIFU therapy exhibited a pronounced improvement of memory performance and increases in neuronal and dendritic length (Jolesz, 2014). Therefore, forthcoming studies need to be done using focused ultrasound for Alzheimer's patients.

### Essential Tremor

The FDA granted approval for MR-guided focused ultrasound mediated unilateral lesioning for the treatment essential tremor (ET) in 2016. ET is a condition where current medical therapy is mostly insufficient for patients will severe and disabling tremor. Medical experience has shown reduction of tremor through targeting the ventral intermediate nucleus of the thalamus (VIM) with either sterotactic lesioning or deep brain stimulation. HIFU can be used to form a brain lesion in less invasive manner compared to surgical therapy of deep brain stimulation (Fishman, 2017).

The Elias et al. describes a randomized trial of focused ultrasound thalamotomy for ET. In this trial, 76 patients with medication-refractory ET were included and controlled using a sham procedure. The trial exhibited significantly reduced hand tremor at 3 months with tremor scores improved by 47% and this effect was perceived for 12 months through follow up. Side effects of the HIFU therapy included sensory and gait disturbances (Elias et al., 2016). This study and FDA approval have strong implications for future treatments of neurological conditions.

### Parkinson's Disease

The success of MR-guided focused ultrasound has encouraged investigators to target the VIM in tremor-dominant Parkinson's disease (PD) (Lee et al., 2019). A clinical trial conducted by Bond et al. described 27 patients with tremor-dominant PD improving 62% at the 3 month assessment on a clinical rating scale succeeding focused ultrasound thalamotomy (Bond et al., 2017). This trial confirmed the use of HIFU in ET and is considered a pilot study for HIFU use in PD. Another investigation by Martinez-Fernandez et al. investigated the safety and preliminary efficacy of MRI-guided focused ultrasound unilateral subthalamotomy in 10 patients with marked asymmetric Parkinsonism. In this study there was an improvement of 53% from baseline to 6 months in the off-medication state and an improvement of 47% in the on-medication state (Martínez-Fernández et al., 2018). Overall, the ultrasound therapy was well tolerated and modestly achieved improvement in motor features of PD. With these examples and other categories of PD there is evidence of focused ultrasound ablative therapy as an alternative to deep brain stimulation. These trials and others lay a strong foundation to more randomized controlled trials with larger cohorts to widen standard treatment options of PD.

### Ultrasound for Antimicrobial Therapy

Bactericidal effect of ultrasound is very well-known (Yu et al., 2012; Kobayashi et al., 2014; Cai et al., 2017; Spiteri et al., 2017). The acoustic cavitation effect of ultrasound is believed to be involved in damaging the microorganism. The acoustic cavitation can generate mechanical forces such as shock waves, shear forces, and microjets which damage microorganisms (Ashokkumar, 2011). Another mechanism of bactericidal action of ultrasound induced cavitation is generation of a few free radicals in aqueous medium, which are OH^.^ and H^.^ because of the decomposition of the H_2_O molecule. These radicals modulate the cellular construct of the bacteria, which results into the retardation of the bacterial action (Spiteri et al., 2017). The cavitation effect can also be employed for the prevention of biofilm formation on implant surface inside the body. In the antibiotic embedded implants, the release of antibiotic can be enhanced by exposure to ultrasound. Ultrasound radiation pressure and the stable cavitation will generate multidirectional acoustic microstreams, which in turn produce a high shear stress to increase the release and delivery of embedded antibiotic from implants surfaces (Cai et al., 2009). Low frequency focused ultrasound is a promising method to enhance the antibiotic action on bacteria as it has advantages such as capability of treating deep tissue targets without surrounding tissue damage (Cai et al., 2017). However, literature also suggests that the low intensity ultrasound alone is not effective for complete elimination of bacteria, but the combination of low intensity ultrasound and antibiotics has more potential for antibacterial activity (Yu et al., 2012).

More recently, Liu et al. demonstrated the synergistic effectiveness of sonodynamic therapy and immunotherapy (sono-immunotherapy) against one of the difficult to manipulate “methicillin-resistant *Staphylococcus aureus* (MRSA)” bacterial strain. The approach not only kills bacteria but also kills bacteria-associated virulence, therefore termed the study as “one arrow two hawks: a dual approach to eliminating bacterial infection.” Their study also reported that the antimicrobial sonodynamic therapy (SDT) works with the help of sonosensitizers to produce ROS, which are highly lethal for all bacteria without causing resistance. However, a single SDT is not always effective for complete bacterial eradication. Therefore, antivirulence therapy was utilized in this study in combination with SDT. The antivirulence treatment specially deactivated bacterial pathogenicity by nullifying their virulence factors. In this way, the antvirulent technique also combats immunosuppression and preserves natural immune protection from virulence destruction. In addition, this therapy without use of antibiotic will be gentle on gut microbiota, without causing resistance to antibiotics and become less cross resistant to each other (Pang et al., 2019).

### Antibacterial Chemotherapy for Biofilms

Bacterial biofilms are one of the most common reasons for contamination of various medical and biological areas. The existence of bacterial biofilm are major issues from oral biofilms in the mouth to biofilm formation on medical devices (Vyas et al., 2019). Biofilms are usually very tolerant and show resistance to conventional antimicrobial agents like antibiotics. The more complex biofilm structure as compared to single bacterial organisms poses difficulty for antimicrobials to diffuse through (Bjarnsholt, 2013; Wu et al., 2015). Within the biofilm structure, bacteria are encased in a self-produced extracellular matrix. The matrix comprises of extracellular polymeric substances (EPS) that in combination with carbohydrate-binding proteins, flagell, pili, adhesive fibers and extracellular DNA serve as a stable platform/scaffold for the three-dimensional biofilm formation. The matrix is self-sufficient, as within the matrix nutrients are trapped and water is efficiently retained. Enzymes released by the bacteria can alter the EPS make up in response to alter nutrient obtainability. Therefore, the conditions can tailor biofilm architecture to the specific environment. As a results of these favorable conditions, the structural constituents of the matrix becomes thoroughly hydrated and strong due to high tensile strength that keeps bacteria encased within the biofilm structure (Kostakioti et al., 2013). The removal of bacterial biofilm without causing damage to surrounding tissues remains the goal for ultrasound assisted therapy. The current treatments with antibiotics are not effective for removal of biofilm, as the antibiotics are effective to only metabolically active bacteria. In other words, the action of antibiotics may be antagonized by local conditions around bacterial biofilm caused by accumulation of waste products around the biofilm (Stewart and Costerton, 2001).

It has been recently proposed that the use of antimicrobials followed by physical biofilm disruption (with the help of shear stresses of ultrasound) could be an effective strategy for biofilm disruption and management (Koo et al., 2017). The mechanical energy associated with ultrasound can pull out and kill biofilms because of cavitation and acoustic streaming mechanisms associated with ultrasound. It is more effective than antimicrobial agents as it has less likelihood of developing resistance and reoccurrence of the biofilm.

## Design Considerations for a Research Therapeutic Ultrasound System

Some design considerations for a system supporting therapeutic ultrasound research are explored. These systems can be designed around a custom-designed ultrasound system or a clinical ultrasound system. Clinical systems generally have a more limited range of configuration parameters and provide less access to custom software control. In this regard, it is desirable to use a custom-designed ultrasound system purposely-built for research or a clinical system in which special access has been granted to enable more detailed control of the operating parameters. In either case, several provisions are desirable depending upon the research application. In these design considerations, we explore both *in vitro* and *in vivo* experimental conditions.

To begin, we discuss system calibration. No matter the tissue targeted, it is always beneficial to explore and validate the acoustic beam properties experimentally. It can be performed in conjunction with numerical simulations and phantom measurements. System calibration generally requires a robotic, motorized two-axis, or three-axis stage to systematically move a calibrated measurement hydrophone to record the resulting acoustic field under specific testing conditions. In addition to system calibration, the motorized stage may also enable calibrated *in vitro* ultrasound stimulation. In this case, a petri dish may be systematically moved to ensure appropriate exposure of the target medium to the acoustic stimulation. With regard to *in vitro* vs. *in vivo* experiments, the focal depth will likely require modification, with shorter depths required for *in vitro* conditions and longer depths required for *in vivo* conditions.

We now consider transducers whose properties will be chosen for specific applications. With regard to ultrasound stimulation, often a focused beam will be desired. In this case, either a single-channel transducer with a fixed focus may be utilized or a multi-element transducer array with more flexible focus options may be used. The difference between these options should be carefully considered. The single-channel transducer will certainly be less costly, but will necessarily require relative motion of the transducer and tissue to enable movement of the focal region. A multi-element transducer array, on the other hand, enables one to move the focal region within limits by steering the acoustic beam. The offered additional flexibility may be beneficial and desirable, depending on the specific application. A further consideration with regard to transducer arrays is the geometrical configuration of the array, e.g., linear, planar or an annular ring. Planar stimulation over a relatively wider region may be especially useful for *in vitro* testing.

With regard to *in vivo* experimental conditions, it is often useful to combine either a HIFU or LIFU system with additional imaging modalities including standard ultrasound imaging. Standard ultrasound imaging enables passive cavitation imaging which may be useful for therapeutic applications involving the cavitation of microbubbles. The location of the cavitation can then be estimated. MRI-guided focused ultrasound (MRgFUS) couples magnetic resonance imaging with focused ultrasound stimulation. It enables precise targeting of the acoustic stimulation. For MRgFUS, special transducers are required which do not significantly disturb the magnetic field (Speicher et al., 2015).

Here in Table 2, we have summarized the ultrasound parameters like frequency and intensity for *in vivo* and *in vitro* lab studies. The studies include the application of ultrasound for disruption of nanoparticles with ultrasound and ultrasound induced sonoporation effect.

**Table 2:** Parameters for designing of ultrasound machine for *in vivo* and *in vitro* lab studies.

	**Purpose**	**Frequency and intensity**	**Ultrasonic processor**	**Reference**	**Range**
**A. TRIGGERED DRUG RELEASE FROM NANOPARTICLES**					
1	Disruption of liposomes under mild hyperthermia (thermosensitive)	Frequency: 1.0 MHz Intensity: 1981.6 W/cm^2^	Therapy Imaging Probe System, Philips Research, Briarcliff Manor, NY	Park et al., 2013	Frequency 20-kHz−7.5 MHz Intensity: 1.5–5.9 W/cm^2^ up to 1981.6 W/cm^2^ for hyperthermic nanoparticle disruption
2	Release of drugs from liposomes *in vivo* using LFUS. (Low Intensity focused Ultrasound)	Frequency: 20-kHz Intensity: 5.9 W/cm^2^	VC400, Sonics & Materials, Newtown, CT	Schroeder et al., 2009a	
3	Mesoporoussilica composite for effective ultrasound triggered smart drug release *in vivo*	Frequency: 20–50 kHz Intensity: 1.5 W/cm^2^	NA	Jafari et al., 2019	
4	Tumor-penetrating codelivery of siRNA and paclitaxel with ultrasound-responsive nanobubbles	Frequency: 1 MHz Intensity: NA	Therapeutic US system (DCT-700, WELLD, Shenzhen, China)	Yin et al., 2014	
5	Ultrasound-sensitive siRNA-loaded nanobubbles formed by hetero-assembly of polymeric micelles and liposomes	Frequency: 1 MHz, Intensity: 2.0 W/cm^2^	Self-made therapeutic US system (Institute of Ultrasound Imaging, Chongqing Medical University	Yin et al., 2013	
6	PLGA nanoparticles for ultrasound-mediated gene delivery in solid tumors *in vivo*	Frequency: 7.5 MHz	Diagnostic ultrasound system 3535 (Bruel and Kjer, Denmark)	Chumakova et al., 2008	
7	Ultrasound-mediated gene transfer (sonoporation) *in vitro* and prolonged expression of a transgene *in vivo*	Frequency: 40 kHz Intensity: 1.9 W/ cm2	Sonidel SP 100 sonoporation device (Sonidel Ltd., Ireland)	Li et al., 2009	
**B. SONOPORATION** ***in vivo***					
8	Sonoporation enhances liposome accumulation and penetration in tumors with low EPR	Frequency: 16 MHz Intensity: 0.9 W/cm^2^	VisualSonics Vevo2100 imaging system (Fujifilm Sonosite, The Netherlands)	Theek et al., 2016	Frequency: 1–16 MHz Intensity: 0.4–to 2.0 W/cm^2^
9	Multiparameter evaluation of *in vivo* gene delivery using ultrasound-guided, microbubble-enhanced sonoporation	Frequency: 1.4 MHz	Siemens Antares system (Siemens Health Care, Inc., Ultrasound Division, Mountain View, CA, USA)	Shapiro et al., 2016	
10	Combination of chemotherapy and photodynamic therapy for cancer treatment with sonoporation effects	Intensity: 2.0 W/cm^2^	NA	Lee et al., 2018	
11	Ultrasound-responsive polymeric micelles for sonoporation-assisted site-specific therapeutic action (Wu et al., 2017)	Frequency: 1.90 MHz Intensity: 0.4 W/cm^2^	Planar transducer (Institution of Applied Acoustics, Shaanxi Normal University)	Wang et al., 2013; Wu et al., 2017	
12	Epidermal growth factor receptor-targeted sonoporation with microbubbles enhances therapeutic efficacy in a squamous cell carcinoma model	Frequency: 1 MHz Intensity: 2.0 W/cm^2^	Sonitron 2000 sonicator (Rich Mar Inc., Inola, OK, USA).	Hirabayashi et al., 2017	
13	Dual-targeted and pH-sensitive doxorubicin prodrug-microbubble complex with ultrasound for tumor treatment (Luo et al., 2017)	Frequency: 1 MHz Intensity: 2 W/cm^2^	US system (DCT-700, WELLD, Shenzhen, China). (Yin et al., 2014)	Yin et al., 2014; Luo et al., 2017	
***C. In vitro*** **STUDIES**					
	Sonoporation	•Less than 100 kHz, although frequencies up to 16 MHz have been investigated (ter Haar, 2007) • Achibana et al. were the first to image cells with pores by SEM after exposure to low frequency ultrasound of 255 kHz (Lentacker et al., 2014) • Low-frequency (24 kHz) (Keyhani et al., 2001)	•Intensity: 0.9–1.8 W/cm^2^ (Khayamian et al., 2018) • Low acoustic intensity, consisting of 0.5, 1.0, and 1.5 W/cm^2^ (Shi et al., 2017) • 1.5 W/cm^2^ • 0.9W/cm^2^ (Theek et al., 2016) • 0.23 W/cm^2^ (Yu and Xu, 2014)	•Frequency: 3.33 kHz−16 MHz • Intensity: 0.23–1.8 W/cm^2^	
		•Ultrasonic stimulation was carried out for 2 s with frequency of 20 kHz (Khayamian et al., 2018) • 2 MHz (Helfield et al., 2017) • 1 MHz (Shi et al., 2017) • 1 MHz • 16 MHz (Theek et al., 2016) • 1-MHz (Liao et al., 2018) • 3.33 kHz (Bhutto et al., 2018) • 180 kHz (Yu and Xu, 2014)		
	Disruption of nanoparticles	•850 kHz (Stavarache and Paniwnyk, 2018) • 3.3 MHz (Papa et al., 2017) • 1 MHz (Baghbani et al., 2017)	•3.19W (Stavarache and Paniwnyk, 2018) • 2.2 W/cm^2^ (Papa et al., 2017) • 2 W/cm^2^ (Baghbani et al., 2017)	•Frequency: 840 kHz−3.3 MHz • Intensity: 2–3.1 W/cm^2^	
	Enhancing penetration of nanoparticles through tumor (High intensity)	•1.5 MHz (Lee et al., 2017) • 1 MHz (Wang S. et al., 2012) • 1.5 MHz (Han et al., 2017) • 3 MHz (Frazier et al., 2016) • 1.16 MHz (Zhou et al., 2016) • For liposomes in the range of 20 kHz−16 MHz (Dragicevic and Maibach, 2018)	•5 and 20 W/cm^2^ (Lee et al., 2017) • 2685 W/cm ^2^ (Wang S. et al., 2012) • 10 W/cm^2^ (Han et al., 2017) • 816–1,411 W/cm^2^ (Frazier et al., 2016) • 705–900 W/cm^2^ (Zhou et al., 2016)	•Frequency: 1.5–16 MHz • Intensity: 5–2,685 W/cm^2^	

## Ultrasound Parameters

The combination of certain ultrasound parameters including frequency, intensity, mechanical index (MI), and duration of ultrasound exposure can influence the efficacy of ultrasound-mediated drug delivery.

### Therapeutic Ultrasound Transducers/Devices

Ultrasound can be exposed either by using plane wave, non-focused transducers, or focused transducers. Typically, non-focused transducers are employed for achieving physical effects of ultrasound applications and for enhancing transdermal delivery by the process called as sonophoresis. Whereas, in focused ultrasound, radiations can be focused onto a very small area, which leads to an increase the intensity to a great extent and therefore named as high intensity focused ultrasound (HIFU). Focused beams are generated with specially designed spherically-curved transducers, it permits deeper permeation and deposition of energy deep inside the body (Zhang Y. R. et al., 2019). The ultrasound radiation propagates through the skin and other tissues on its way to the target over a large area creating comparatively low spatial intensities and generating no damage (Mullick Chowdhury et al., 2017). At the focus, however, intensities can be 3–4 orders of magnitude higher than at the transducer surface. Targeting of HIFU beam to specific tissues, organs and tumors may be carried out using different imaging modalities: diagnostic (B-scan) ultrasound (Xenariou et al., 2007), and magnetic resonance imaging (MRI) (Chen et al., 2017). Recent studies have also indicated that computed tomography (CT) and optical 3D tracking (Schoellhammer et al., 2017) can also be used for guiding HIFU exposures; however, these imaging modalities have not yet been incorporated into commercial HIFU devices. The advantages of using extra-corporeal HIFU exposures, for example for tumor ablation, compared to more invasive surgical procedures, are many-fold, and include limited blood loss and infection, elimination of scar formation, and a decreased risk of other complications. Tumor ablation with HIFU can also be provided on an outpatient basis, where cost and recovery times are significantly reduced in comparison to other existing techniques, such as radio frequency ablation, laser, and cryoablation (Ensign et al., 2012).

### Frequency

The average ultrasound frequency utilized in drug delivery varies from kilohertz to Megahertz levels. It depends on the type of cells and the model animal selected for the particular experiment. Typically, the ultrasound frequency used for drug delivery and other therapeutic applications, is lesser than diagnostic purpose. The higher levels of frequencies may account for cavitation effect which arises from short-pulse, low-duty cycle diagnostic ultrasound (Zacchè et al., 2015). The lower ranges of ultrasound frequencies has capabilities to penetrate deeply situated tissues as they have low attenuation effect which can ensure the desired therapeutic effect without causing attenuation related side effects. The frequency range used for microbubble and ultrasound assisted treatments are also subjected to type of microbubbles used in a particular experiment. It is because the ultrasound frequency near to or equal to resonance frequency of the microbubbles enhances stable microbubble cavitation (Hu et al., 2010; Qin et al., 2018). On the other hand, high frequency has high resolution but low tissue penetration. For example, transdermal drug delivery (TDD), which includes an increase in skin permeabilization, requires ultrasound waves of 55 KHz]. Intravascular thrombus dissolution requires 2.2 MHz. Even higher level of frequencies are used for cancer therapy using hyperthermia 1–5 MHz frequency. The ultrasound frequency used for the therapeutic purposes is lower than that for diagnostic purposes (Joshi and Joshi, 2019). Lower frequencies of ultrasound covers the frequencies lower than 1 MHz, whereas, erate and high acoustic frequencies are in the range of 1–5 and 5–10 MHz, respectively (Du et al., 2011). Table 3 summarizes the use of nanoparticles in combination with ultrasound to enhance efficacy of the treatment.

**Table 3:** Ultrasound frequencies used for various medical application.

**Frequencies**		**Applications**	**References**
>20 KHz	Audible region	NA	
19.5, 22.5, 42 KHz	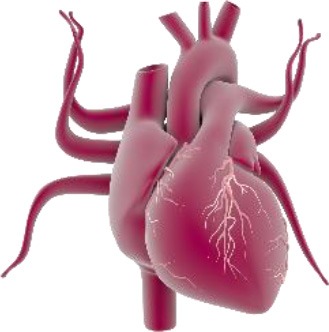	Angioplasty	Siegel et al., 1994; Goyen et al., 2000; Wylie et al., 2009
20 KHz-1 GHzs	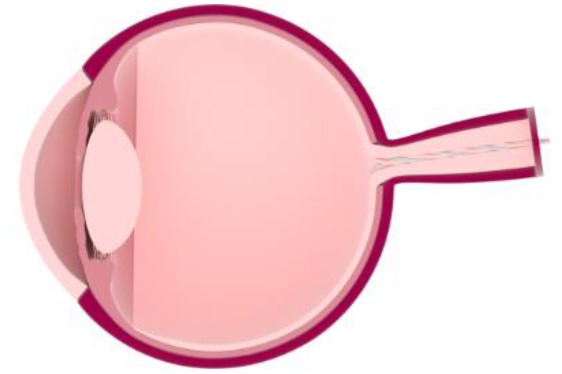	Ocular drug delivery	Zderic et al., 2002; Hariharan et al., 2017; Lafond et al., 2017
0.25–2 MHz	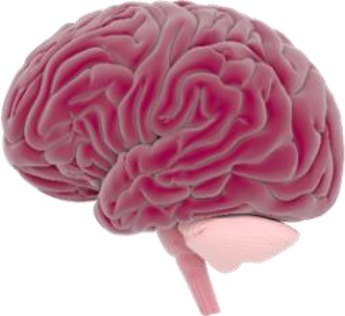	Drug delivery to central nervous system	O'Reilly and Hynynen, 2012
20 kHz−16 MHz	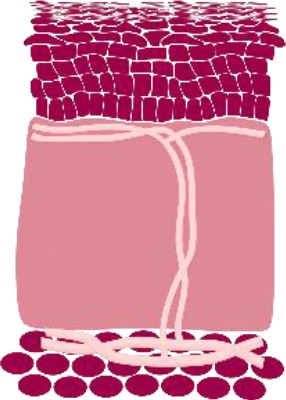	Transdermal drug delivery	Mitragotri, 2017
255 KHz−5 MHz	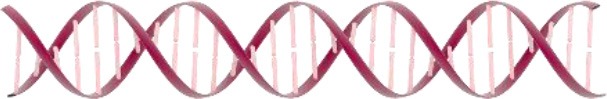	Gene delivery	Yu et al., 2019
1–4 MHz	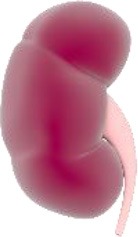	Kidney stone ablation	Ikeda et al., 2016
1 MHZ	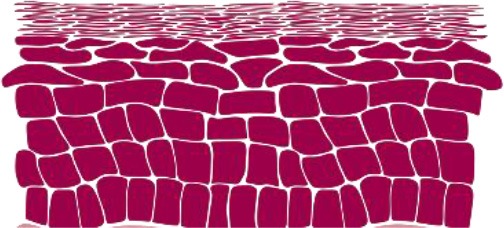	Topical delivery of hydrocartisone	
1.5 MHz−1.0 kHz	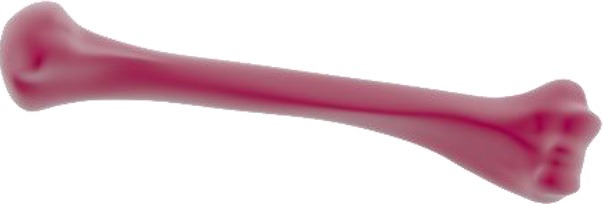	Osteoporosis	Ozdemir et al., 2008
≥1 MHz	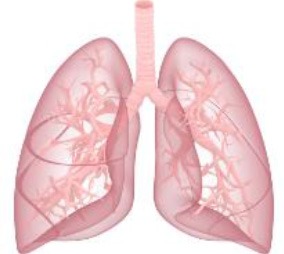	Nebulizer for pulmonary drug delivery	Wiedmann and Ravichandran, 2001
20 kHz	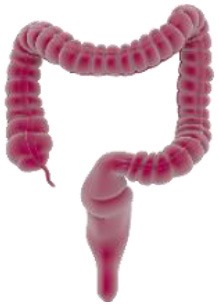	Colon	Schoellhammer and Traverso, 2016
0·96 MHz	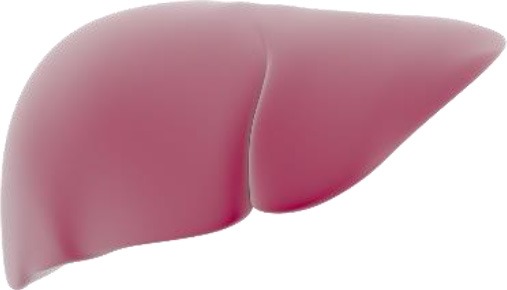	Liver hyperthermia	Lyon et al., 2018
0.5–2 MH	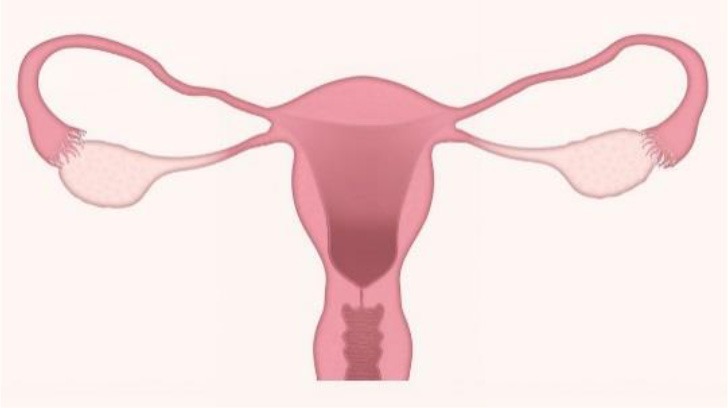	Ablation of uterine fibroids	Fan et al., 2019
<10 MHZ		Diagnosis	

### Intensity

The potential use of high intensity ultrasound (Azagury et al., 2016) is to induce ultrasound associated heating effects that alters the tissue function. Therefore, the FDA has recommended the intensity that causes the heating of tissues less than a 1°C rise in temperature (Azagury et al., 2016; Guan and Xu, 2016; Mullick Chowdhury et al., 2017). Generally, for drug delivery applications intensity range of 0.3–3 W/cm^2^ is used. Higher intensities ultrasound can be employed when the pulse length (pulse cycles/ultrasound frequency) and/or pulse repetition frequency (pulses/sec) values are reduced (Joshi and Joshi, 2019).

### Mechanical Index

The mechanical index (MI) of ultrasound can be explained as the peak negative pressure (in MPa) divided by the square root of center frequency (in MHz). The MI is used as an alternative variable to ultrasound intensity because they are directly proportional to the relevant acoustic pressure. Also, the MI gives an exact estimation of the produced cavitation, greater MI values induce greater cavitation activity. To circumvent undesirable thermal adverse effects in the course of ultrasound treatment, the MI in the range of 0.2 and 1.9 is used, and the FDA has a recommendation of the higher limit of the MI to 1.9 for clinical use of ultrasound to bypass direct tissue harm by ultrasound (Izadifar et al., 2019).

### Treatment Duration

The duration of treatment in ultrasound assisted drug delivery and therapeutic ultrasound should be determined by the time required for the ultrasound to generate a desirable effect without inducing unwanted effects on the body. Moreover, the time course of ultrasound treatment in drug delivery can be influenced by other factors such as the situation and type of tissues undergoing treatment, ultrasound settings (ultrasound intensity being applied, and frequency), as well as the type of microbubbles used (if applicable) (Juffermans et al., 2006). High pressures of ultrasound waves can cause spontaneous inertial cavitation, in some cases longer treatment duration can increase the drug delivery. However, high pressures are associated with undesirable effects on body. In a similar way, for lower pressures, the duration required for stable oscillations of microbubbles should be to be contemplate for achieving the most effective drug delivery because longer treatment duration at low pressures can also induce heating effects. Therefore, the precise treatment time in every research protocol is different for different therapeutic applications, and it has to be optimized for each treatment indication (Schroeder et al., 2009b; Joshi and Joshi, 2019).

## Summary

Ultrasound with its tunable intensity and frequency can be utilized in a safe manner to diagnose and treat a wide range of medical conditions. It has been shown that ultrasound has an immense potential to improve cancer treatment by increasing targeting and accumulation of drugs and genes delivered to tissues (Ng and Liu, 2002; Duvshani-Eshet and Machluf, 2005; Tzu-Yin et al., 2013; Mullick Chowdhury et al., 2016). Ultrasound is considered non-invasive and the ultrasound radiation can be focused onto a very small region, as small as a few millimeters. The focal point of the ultrasound beam can penetrate deep into body allowing very precise thermal ablation of tissues and the enhancement of drug delivery to a selected region during treatment (Ohmura et al., 2011; Chen and Hwang, 2013).

Furthermore, by combining ultrasound with nanoparticles, it is possible to develop on-demand drug delivery system where drug release is triggered with ultrasound energy that disrupts drug-carrying nanoparticles. A combination of ultrasound with nanoparticles to deliver drugs has already been applied for treating Alzheimer's disease, cardiovascular disease, infections and cancer (Wang et al., 2015). Ultrasound-mediated drug delivery takes advantage of the enhanced permeability of cell membrane induced by the ultrasound—sonoporation. Increased permeability permits more effective delivery of drug molecules through cell membrane and through other physiological barriers, e.g., blood brain barrier. Importantly, plasma membrane permeabilization induced by acoustic cavitation is a transient process and the membrane integrity usually returns to its original configuration within seconds (Liu et al., 1998).

Despite ultrasound technology being already in place at virtually every hospital/clinic, its use for therapeutic application has been undervalued for long time. More recently the use of ultrasound with microbubbles was approved by the Food and Drug Administration (FDA) for diagnostic applications (Mullick Chowdhury et al., 2017). Moreover, there are many ultrasound-responsive microbubbles and nanoparticle formulations that are undergoing clinical trials or have been used clinically as the ultrasound contrast agents and for the enhanced ultrasound-triggered drug release application. For example SonoVue, Definity, Optison, and Sonazoid are being utilized as an ultrasound contrast agent and ThermoDox (liposomal doxorubicin) is used to enhance temperature-triggered doxorubicin release induced by magnetic resonance high intensity focused ultrasound (Anselmo and Mitragotri, 2019). The increasing use of ultrasound with microbubbles/nanoparticles in clinics supports future clinical translation of ultrasound-enhanced nanoparticle drug delivery for improving drug delivery and drug therapy in cancer and several other medical conditions.

While there are many benefits of using ultrasound, the impact of the energy of ultrasound radiation on cell integrity raises some concerns. Ultrasound energies higher than the cavitation threshold can disrupt the cell membrane. A high dosage of ultrasound can thermally and sonochemically induce permanent damage to lipid membranes, and cause denaturation of proteins and DNA (Domenici et al., 2013). But these perceived limitations, can be turned into therapeutic applications which include tissue hyperthermia and thermal ablation which are both used to obliterate diseased tissues.

A further limitation is that ultrasound cannot be used in all environments and there is a need to optimize the beam conditions depending on the presence of gas within tissues (e.g., lungs) which is an obstacle for propagation of ultrasound waves. The organ associated movements during the ultrasound treatment procedure is another major problem, as the continuous movements of organs cause distortion of ultrasound beam focal point. Also, while treating deep-seated tumors in the body, there is an issue with the ultrasound beam being attenuated or deflected from hard tissues during its travel to the target area. For example, in the treatment of lung cancer, the presence of both bone (ribcage) and gas may interfere with propagating ultrasound waves. Ultrasound reflection by bone or gas-containing tissue may cause collateral damage and undesired skin burns when high intensity ultrasound is used.

In summary, ultrasound has the potential to improve treatment of cancer and other medical conditions. However, more research and developments are needed for the design of a therapeutic ultrasound system considering the location of the tumor in the body. Also, a better understanding of the underlying mechanisms of the interaction of the ultrasound beam with different cell types is essential.

## Author Contributions

PT designed an outline for the manuscript and wrote most of the parts of the manuscript. PT also contributed toward execution of ideas for drawing all graphical pictures in the manuscript by a graphic designer. RV contributed to writing some sections of the manuscript such ‘ultrasound sensitive nanoparticles', application of HIFU in different types of tumor, and application of therapeutic HIFU in central nervous system disorders. RV also contributed to proof reading of the article. CJ wrote and shared his ideas for the section about design considerations for designing of the ultrasound system for research purposes. WW proofread the manuscript. WC is the corresponding author of the manuscript, supervised, monitored the writing process time to time, and wrote add his inputs to the manuscript during the checking and correcting the manuscript. All authors reviewed the manuscript.

### Conflict of Interest

The authors declare that the research was conducted in the absence of any commercial or financial relationships that could be construed as a potential conflict of interest.
